# Protein Biomarkers of Kidney Injury and Dysfunction: Emerging Evidence on Non-Coding RNAs

**DOI:** 10.3390/diagnostics16213670

**Published:** 2026-11-06

**Authors:** Eduardo Pérez-Campos Mayoral, María Teresa Hernández-Huerta, Laura Pérez-Campos Mayoral, Gabriel Mayoral-Andrade, Héctor Alejandro Cabrera-Fuentes, Carlos Romero Díaz, Gabriel Mayoral Canseco, Jesús Elizarrarás-Rivas, María del Socorro Pina-Canseco, Gabriela Vásquez-Martínez, Margarito Martínez-Cruz, Alma Dolores Pérez Santiago, Miriam Emily Avendaño Villegas, Casimiro Romeo Zarate Aspiros, Luis Otilio Flores León, Romina Fernanda Camarena Colmenares, Ulises Jesús Roldán Trejo, Tania Sinaí Santiago-Ramírez, Juan de Dios Ruiz-Rosado, Eduardo Pérez-Campos

**Affiliations:** 1 Centro de Investigación Facultad de Medicina UNAM-UABJO, Facultad de Medicina y Cirugía, Universidad Autónoma “Benito Juárez” de Oaxaca (UABJO), Oaxaca 68020, Mexico; eperezcampos.fmc@uabjo.mx (E.P.-C.M.); mthernandez@secihti.mx (M.T.H.-H.); lperezcampos.fmc@uabjo.mx (L.P.-C.M.); gmayoral.fmc@uabjo.mx (G.M.-A.); hector.cf@tectijuana.edu.mx (H.A.C.-F.); mpina.cat@uabjo.mx (M.d.S.P.-C.); aspiros59@hotmail.com (C.R.Z.A.); foll050720.fmc@uabjo.mx (L.O.F.L.); cacr040505.fmc@uabjo.mx (R.F.C.C.); ulisesroldant@gmail.com (U.J.R.T.); 2 Secretaría de Ciencia, Humanidades, Tecnología e Innovación (SECIHTI), Facultad de Medicina y Cirugía, Universidad Autónoma “Benito Juárez” de Oaxaca, Oaxaca 68020, Mexico; 3 División de Estudios de Posgrado e Investigación, Tecnológico Nacional de México/ Instituto Tecnológico de Tijuana, Tijuana 22414, Mexico; 4 R&D Group, Vice Presidency Scientific Research & Innovation, Imam Abdulrahman Bin Faisal University (IAU), Dammam 31451, Saudi Arabia; 5 División de Estudios de Posgrado e Investigación, Tecnológico Nacional de México/Instituto Tecnológico de Oaxaca, Oaxaca 68030, Mexico; carlos.romero@itoaxaca.edu.mx (C.R.D.); dr.gabrielmayoral@gmail.com (G.M.C.); mcruz@itoaxaca.edu.mx (M.M.-C.); aperez_santiago@hotmail.com (A.D.P.S.); e_mily_3@hotmail.com (M.E.A.V.); 6 Instituto Mexicano del Seguro Social (IMSS) Bienestar Servicios de Salud de Oaxaca, Oaxaca 68000, Mexico; dr.jesuselizarraras@gmail.com; 7 Facultad de Medicina y Cirugía, Universidad Autónoma “Benito Juárez” de Oaxaca, Oaxaca 68020, Mexico; 8 Kidney and Urinary Tract Research Center, Abigail Wexner Research Institute, Nationwide Children’s Hospital, Columbus, OH 43215, USA; gabriela.vasquezmartinez@nationwidechildrens.org; 9 Dirección de la División de Investigación y Desarrollo Científico, Benemérita Universidad de Oaxaca, Oaxaca 68000, Mexico; ttanniaramirez@gmail.com

**Keywords:** acute kidney injury, chronic kidney disease, diagnostic biomarkers, prognosis, non-coding RNA, microRNA, urinary biomarkers, clinical validation

## Abstract

**Background:** Kidney biomarkers address different clinical questions, and their accuracy depends on the population, sample, timing, and outcome. **Objective:** To organize selected evidence on protein biomarkers of kidney injury and dysfunction and examine emerging clinical evidence on non-coding RNAs (ncRNAs). **Methods:** This structured comparative narrative review used PubMed and Google Scholar, with searches through 19 September 2026, supplemented by targeted source verification and literature updating during revision in October 2026. Published estimates were summarized descriptively, no new meta-analysis was performed. Filtration assessment, AKI detection, persistence, disease activity, and RNA discovery or validation were considered separately. **Results:** Cystatin C and proenkephalin provide filtration-related information; KIM-1, NGAL, L-FABP, and NAG address tubular injury or associated outcomes. Urinary IL-18 and serum IL-8 have limited stand-alone diagnostic value in pediatric AKI. [TIMP-2]×[IGFBP7] reflects cellular stress and near-term AKI risk. CCL14 concerns persistent AKI. uDKK3, VCAM-1, and serum cathepsin S have evidence specific to their applications. Urinary HBP and plasma 8-isoprostane describe complementary infection and oxidative-stress contexts. More recent syntheses revise previously reported estimates for KIM-1 and NGAL. Selected ncRNA studies show promising discrimination, but their populations, clinical contrasts, and validation stages differ. **Conclusions:** Organizing biomarkers by clinical question connects their biological information with distinct diagnostic and prognostic applications. Protein–ncRNA combinations offer a direction for prospective research, with clinical adoption requiring independent validation and added value beyond conventional assessment.

## 1. Introduction

Kidney injury and kidney dysfunction are related but are not equivalent diagnostic targets. Serum creatinine and urine output remain central to the assessment of acute kidney injury (AKI), yet they describe functional change rather than identify a specific cellular lesion [1]. Serum creatinine is also affected by its production, distribution, and elimination, particularly when kidney function is not at steady state [2]. These limitations have motivated evaluation of complementary markers of damage, stress, inflammation, and AKI persistence [2,3,4]. Urinary biomarkers are also investigated for chronic kidney disease (CKD) detection and progression [5].

A similar distinction is relevant in CKD. A marker associated with reduced estimated glomerular filtration rate (eGFR), one associated with active inflammation, and one associated with subsequent eGFR loss answer different questions. They cannot be interpreted as equivalent tests merely because each is associated with kidney disease. Reviews of urinary biomarkers and AKI pathophysiology emphasize the separation between biological mechanisms and intended clinical applications [5,6,7].

The biomarkers considered here span several biological domains. KIM-1, NGAL, N-acetyl-β-D-glucosaminidase (NAG), and liver-type Fatty Acid-Binding Protein (L-FABP) have been investigated in relation to tubular injury. Cystatin C and proenkephalin provide filtration-related information, whereas urinary [TIMP-2]×[IGFBP7] is associated with cellular stress and AKI risk [4]. Inflammatory markers include interleukin (IL)-18 and IL-8/CXCL8; urinary CCL14 has been evaluated for persistence after AKI has developed [3,4]. Urinary Dickkopf-related protein 3 (uDKK3) has also been associated with subsequent eGFR loss in CKD [8]; these targets require separate interpretation.

Comparing these markers requires attention to the intended use and study setting; published syntheses describe heterogeneity in populations, AKI definitions, sampling times, assays, and thresholds [2,3]. Therefore, their pooled results should not be interpreted as a universal ranking. Non-coding RNAs (ncRNAs) add a different level of biological information: microRNAs (miRNAs) and long non-coding RNAs (lncRNAs) have been implicated in kidney injury, inflammation, fibrosis, and repair [9,10,11,12].

This narrative review examines the clinical applications of protein biomarkers of kidney injury and dysfunction, along with emerging evidence on ncRNAs. Reported performance is interpreted in relation to intended use, study population, study design, specimen type, and validation stage. For ncRNAs, experimental findings on kidney injury and repair are distinguished from human evidence of diagnostic or prognostic value. Urinary HBP and the nonprotein analyte plasma 8-isoprostane are discussed as complementary examples of infection and oxidative stress, respectively, to clarify the limits of interpreting an abnormal biomarker result as evidence of structural kidney injury. The resulting framework links each biomarker to its clinical question and identifies where protein–ncRNA combinations warrant prospective evaluation.

## 2. Scope and Methods

This structured comparative narrative review organizes selected evidence by biological domain, intended clinical use, and stage of validation. The original PubMed and Google Scholar searches covered publications through 19 September 2026. Marker-specific searches were combined with terms for acute kidney injury, chronic kidney disease, diabetic kidney disease, lupus nephritis, urinary tract infection, sensitivity, specificity, receiver operating characteristic, AUC, and diagnostic accuracy. During revision, targeted searches and checks of PubMed records, publisher full texts, and the relevant regulatory document were completed on 23 October 2026 to address omitted biomarkers, identify newer KIM-1 and NGAL syntheses, and verify disputed estimates. This targeted update was not a new exhaustive systematic search.

Selection was purposive and question-based; clinical evidence was considered for filtration assessment, AKI detection or risk, AKI persistence, CKD progression, and disease-specific activity. Systematic reviews and meta-analyses informed the quantitative summaries where applicable, including estimates of AKI diagnostic accuracy, while primary studies supplied complementary disease-specific, longitudinal, or RNA validation evidence. Established comparators such as cystatin C and additional tubular markers such as L-FABP and NAG were retained even when their selected evidence could not be represented by a single sensitivity–specificity–AUC profile. Evidence concerning persistent AKI, CKD progression, and disease-specific activity was interpreted according to the respective outcome and follow-up period.

Infection-associated and oxidative-stress markers were considered within their specific clinical contexts, without treating their reported performance as equivalent to discrimination of structural kidney injury. Human ncRNA studies were assessed according to the clinical question and validation stage, whereas experimental studies were used to discuss mechanisms and kept separate from human diagnostic evidence. The review is not an exhaustive catalogue, and a marker’s omission does not establish a lack of biological or clinical relevance.

For each selected estimate, the marker–specimen–population–outcome combination was the unit of comparison, with interpretation also accounting for study design. Selected thresholds and reported uncertainty intervals were checked against the source publication. Confidence intervals (CI) and intervals labelled as credible intervals (CrI) by the source are identified separately. No missing sensitivity or specificity was inferred from an AUC, and no new pooled estimates or diagnostic metrics were calculated. Historical KIM-1 and NGAL estimates were retained in the text to explain differences from newer syntheses and were not combined into a new estimate. These descriptive comparisons do not constitute a diagnostic algorithm or provide direct evidence that one marker outperforms another. Differences in populations, samples, thresholds, and reference definitions limit direct comparisons across studies [2,5].

## 3. Biomarkers for AKI Detection and Risk Assessment

### 3.1. KIM-1 and NGAL

KIM-1 is associated with proximal tubular injury [13]. The 2014 meta-analysis reported sensitivity of 74% (95% CI, 61–84%), specificity of 86% (74–93%), and AUC of 0.86 (0.83–0.89) [14]. A 2025 adult synthesis reported urinary KIM-1 sensitivity of 73% (95% CrI, 67–78%), specificity of 75% (70–80%), and AUC of 0.81 (0.77–0.84) [15]. Table 1 uses the newer urinary estimate. Differences in study selection, populations, timing, and analytical methods prevent interpreting the change as a temporal deterioration in test performance; neither estimate establishes a universal confirmatory or rule-out role.

NGAL has been evaluated in pediatric critical illness and cardiac surgery [21,22]. The 2017 pediatric urinary synthesis reported sensitivity of 76% (62–85%), specificity of 93% (88–96%), and AUC of 0.94 (0.92–0.96) [23]. The 2023 synthesis reported urinary sensitivity of 83% (95% CI, 78–87%), specificity of 81% (77–85%), and AUC of 0.89 (0.86–0.91) [16]. Table 1 uses this newer pediatric estimate. These differing estimates reflect different evidence sets and are not head-to-head comparisons. They should not be transferred to plasma measurements or unselected adult populations. In an adult septic cohort, plasma NGAL was elevated even without AKI, whereas urinary NGAL was less affected and discriminated subsequent AKI better [24].

### 3.2. Cystatin C and Proenkephalin

Serum cystatin C is a filtration marker used to estimate GFR; creatinine–cystatin C equations have been developed and validated to assess kidney function against measured GFR [25]. This application differs from detecting a structural tubular lesion or predicting incident AKI. eGFR accuracy against measured GFR cannot be converted into universal AKI sensitivity or specificity. Table 2 therefore includes cystatin C as a functional comparator and keeps its evidence separate from the acute-event estimates in Table 1.

Proenkephalin (PENK/penKid) is primarily considered a kidney-function biomarker rather than a direct measure of structural tubular damage; its clinical investigation includes assessment of kidney function and severe sepsis or shock [28,29]. The selected meta-analysis reported sensitivity of 69% (62–75%), specificity of 76% (68–82%), and AUC of 0.77 (0.73–0.81) [17]. The moderate pooled discrimination argues against treating PENK as a stand-alone diagnostic test for all forms of AKI. The same meta-analysis identified an estimated optimal cutoff around 57.3 pmol/L, but its pooled accuracy estimates summarize studies with different thresholds. They should not be presented as if they were all measured at a single, universally validated operating point. Consider pre-existing disease and the clinical setting when applying the pooled estimates [17].

### 3.3. Cell Stress and Near-Term Risk

The product of urinary TIMP-2 and IGFBP7 has been investigated as a cell-cycle-arrest-associated signal for AKI risk. Discovery and clinical-adjudication studies support the distinction between identifying patients at increased near-term risk and diagnosing an already established lesion [30,31]. The selected meta-analysis reported sensitivity of 79% (72–84%), specificity of 70% (62–76%), and AUC of 0.81 (0.78–0.84) [18]. Neither marker should be described as confirming tubular injury independently of its clinical context. In the cardiac-surgery study, a panel of injury biomarkers provided only moderate AKI discrimination, and the KIM-1 and L-FABP associations were not independent of the other injury markers [26]. The functional–damage distinction remains useful, but it is a framework for interpretation and not proof that all proposed combinations are clinically validated [32].

Regulatory status applies to a particular assay and intended use. The U.S. Food and Drug Administration (FDA) decision summary for VIDAS NEPHROCHECK specifies use with clinical evaluation in ICU patients aged 21 years or older who currently have, or have had within the preceding 24 h, acute cardiovascular or respiratory compromise, to assess the risk of moderate or severe AKI within 12 h. The document specifies central laboratory use and excludes point-of-care use [33]. These conditions should not be generalized to all platforms, patient populations, or biomarker combinations.

### 3.4. L-FABP and NAG

L-FABP and NAG provide additional evidence about tubular injury. Urinary L-FABP has been evaluated after cardiac surgery and in broader analyses of hospital-acquired AKI [2,26]. Its interpretation depends on the sampling interval and whether concentrations are reported directly or normalized to urinary creatinine. Table 2 includes the creatinine-normalized L-FABP result from the 2022 synthesis as an application-specific example; it should not be treated as interchangeable with unnormalized L-FABP or a universal threshold.

NAG is a lysosomal enzyme studied as a urinary indicator of tubular injury [4]. In a prospective study of 201 hospitalized patients with established acute renal failure, higher urinary NAG activity was associated with dialysis requirement or in-hospital death [27]. This is prognostic evidence within established illness and does not, by itself, establish performance for detecting new AKI. Table 2 retains this distinction rather than assigning an unverified diagnostic operating point.

### 3.5. Urinary IL-18 and Serum IL-8

IL-18 and IL-8/CXCL8 are distinct inflammatory analytes. The pediatric meta-analysis by Yousefifard et al. evaluated both urinary IL-18 and serum IL-8 [20]. Urinary IL-18 showed sensitivity of 64% (95% CI, 32–87%), specificity of 75% (62–85%), and AUC of 0.77 (0.74–0.81); serum IL-8 showed sensitivity of 53% (34–72%), specificity of 79% (60–91%), and AUC of 0.72 (0.68–0.76) [20]. Table 1 includes both separately. Their different specimens and uncertainty intervals limit direct comparison, and the low sensitivities do not support reliable stand-alone exclusion of AKI in the studied pediatric settings.

### 3.6. uDKK3 in Acute-Event Prediction

uDKK3 has been assessed for AKI prediction in contrast-related and cardiac-surgery settings. The selected meta-analysis reported sensitivity of 55% (41–68%), specificity of 80% (70–87%), and AUC of 0.74 (0.70–0.77) [19]. These figures concern acute-event prediction. They do not estimate discrimination for future CKD progression or histological fibrosis; we consider those applications separately in Section 4.2.

## 4. Persistent AKI, CKD Progression, and Disease-Specific Applications

### 4.1. CCL14 and Persistent AKI

Urinary CCL14 addresses prognosis after AKI has developed. RUBY enrolled ICU patients with KDIGO stage 2 or 3 AKI and evaluated persistent stage 3 AKI lasting at least 72 h [34]. The 2023 meta-analysis reported sensitivity of 81% (95% CI, 72–87%), specificity of 71% (53–84%), and HSROC AUC of 0.84 (0.80–0.87) [35]. Its studies used outcomes ranging from persistence over 48–72 h to non-recovery or dialysis dependence at later follow-up [35]. A broader 2025 synthesis reported a CCL14 AUC of 0.79 (95% CI, 0.75–0.82) [3]; Table 3 identifies these estimates separately. Do not combine them into a single operating point or interpret them as interchangeable predictions of long-term non-recovery.

### 4.2. uDKK3 in CKD and VCAM-1 in Lupus Nephritis

In CKD cohorts, urinary DKK3 was associated with subsequent eGFR loss; the same publication also examined its relationship with tubulointerstitial fibrosis in kidney biopsies [8]. These are separate longitudinal and histological analyses. However, the AUC of 0.74 reported in Table 1 is derived from a meta-analysis of uDKK3 for AKI prediction [19]; it is not an estimate of diagnostic accuracy for chronic fibrosis or of discrimination for future CKD progression. Table 3 includes this progression-related evidence separately. The same analyte can have more than one intended use without sharing a single validated cutoff or performance profile across those uses.

Urinary soluble VCAM-1 has been studied in lupus nephritis activity and treatment response. The active-versus-inactive comparison reported sensitivity and specificity of 74.2% each, with AUC 0.84 (95% CI, 0.75–0.94), at >47.2 ng/mg creatinine [36]. The source table reproduces these percentages but does not provide separate confidence intervals for sensitivity and specificity. These results concern activity classification within lupus nephritis. A systematic review of lupus nephritis biomarkers also identified heterogeneous study definitions and limited multicenter validation [38].

### 4.3. Serum Cathepsin S in Diabetic Kidney Disease

In a cross-sectional study of type 2 diabetes, serum cathepsin S discriminated diabetic kidney disease (DKD) defined by urinary albumin-to-creatinine ratio (UACR) ≥30 mg/g from UACR <30 mg/g, with sensitivity of 71.6%, specificity of 98.8%, and AUC of 0.900 (95% CI, 0.858–0.943) at 827.42 pg/mL [37]. This contrast includes patients above 300 mg/g and is not confined to early DKD. Comparisons with cystatin C also depended on the outcome: cathepsin S had the higher AUC for albuminuria-defined DKD, whereas cystatin C had the higher AUC for reduced eGFR [37]. Cathepsin S was separately associated with lower insulin sensitivity and incident type 2 diabetes (T2D) in elderly men [39]. Such extra-renal associations are relevant to specificity but do not provide the numerical diagnostic evidence for diabetic kidney disease.

## 5. Complementary Infection and Oxidative Stress Contexts

### 5.1. HBP in Urinary Tract Infection

Urinary heparin-binding protein (HBP/azurocidin) has been investigated for urinary tract infection (UTI). In a case–control study of women aged ≥65 years, symptomatic UTI was compared with asymptomatic controls, including women with asymptomatic bacteriuria. The individual-marker AUC was 0.92; at 16.7 ng/mmol urinary creatinine, sensitivity was 86% (95% CI, 75–93%) and specificity was 89% (82–94%) [40]. Earlier adult work included cystitis and pyelonephritis [41]. The estimates in Table 4 concern UTI discrimination and do not establish accuracy for structural renal parenchymal injury.

### 5.2. Plasma 8-Isoprostane and Reduced eGFR

In hypertensive patients, plasma 8-isoprostane showed AUC 0.931 and sensitivity and specificity of 89% at 329 pg/mL for discrimination of eGFR <60 mL/min/1.73 m^2^ [42]. These cross-sectional estimates concern existing dysfunction, not future eGFR decline. Endothelin-1 was measured separately; the reported 8-isoprostane result is not a combined assay [42]. The biological context is oxidative stress in hypertension [43]. Isoprostanes are nonprotein products of lipid peroxidation [44], so this analyte is presented separately from the protein biomarkers.

## 6. Emerging ncRNA Evidence

### 6.1. Human AKI Studies

Serum miR-5100 was evaluated in patients with AKI versus healthy controls, with AUC 0.96, sensitivity 100%, and specificity 83.3% at a study-specific expression cutoff of 0.14 [45]; in companion mouse experiments, delivery of a miR-5100 mimic reduced ischemia–reperfusion injury. Human ROC analysis and the animal intervention address different questions. Neither the healthy-control comparison nor experimental protection establishes discrimination among critically ill patients without AKI or clinical detection before creatinine rises.

Aguado-Fraile et al. studied ten serum miRNAs in a pilot design that included patients with AKI in intensive care and patients undergoing cardiac surgery [46]. Individual-marker AUCs for the ICU comparison were 0.90–1.00; these do not reflect the performance of a single externally validated ten-miRNA classifier. Healthy controls were used for that diagnostic comparison, which limits transferability to critically ill patients without AKI. The preoperative cardiac-surgery analysis addressed a different prediction question. Table 5 separates these analyses and does not assign one sensitivity and specificity to all ten markers.

### 6.2. Human CKD and Diabetic Kidney Disease Studies

Urinary exosomal miR-181a was reported to be reduced in CKD [50]. A separate study developed a urinary model with miR-542-5p, miR-33b-5p, miR-190a-3p, miR-507, and the pseudogene-associated transcript CSAG4. Its development analysis reported AUC 0.9625 (95% CI, 0.9281–0.9969), sensitivity 100%, and specificity 92.5%; a separate RT-qPCR set of 20 CKD patients and 20 healthy controls yielded a reported AUC of 1.00 (95% CI, 1.00–1.00) for the same RNA combination [47]. The publication does not clearly establish external validation of a locked prediction model across assay platforms. These analyses should remain separate, and the small validation set does not establish transportability; CKD-versus-healthy-control discrimination does not establish histological fibrosis staging or prediction of progression. More recent human evidence includes urinary exosomal miR-136-5p in diabetic kidney disease. Zheng et al. reported AUC 0.722 (95% CI, 0.552–0.892), sensitivity 72.2%, and specificity 78.4% at a relative-expression cutoff of 0.141 for DKD versus T2D without DKD [48]. The study extends disease-specific evidence but does not establish a routine diagnostic test; associations with eGFR, albuminuria, or risk categories should not be presented as prospective prediction of CKD progression.

### 6.3. Mechanistic Evidence and Exploratory Clinical Translation

Mechanistic ncRNA findings require separate interpretation. HOTAIR promoted injury through the miR-22/HMGB1 pathway in one experimental sepsis study [51], whereas another rat study reported protection with HOTAIR overexpression, reduced miR-34a, and increased Bcl-2 expression [52]. These findings do not establish a uniformly protective mechanism. RSDR reduced ferroptosis in mouse AKI; the same study measured RSDR in urinary sediment from 69 patients with AKI and 84 non-AKI controls [49]. RSDR alone yielded AUC 0.7452, sensitivity 72.46%, and specificity 72.62%. A logistic model combining RSDR, BUN, serum creatinine, and eGFR yielded AUC 0.864 in internal validation [49]. These are different analyses, and external prospective validation remains necessary; Table 5 reports the individual-marker result.

ncRNAs regulate gene expression and play mechanistic roles in kidney injury and repair [9,10,11,12]. The selected clinical studies use different specimens, including serum, urine, and urinary extracellular-vesicle fractions, with different analytical procedures [46,47,48,50]. A recent review specifically identifies limited methodological standardization and selected study populations as barriers to clinical biomarker use [53]. Therapeutic translation poses additional challenges in delivery, targeting, and safety [54]; these should be distinguished from diagnostic assay validation. Future diagnostic studies should prespecify collection time, reference controls, normalization, and decision thresholds, and test performance in independent populations.

## 7. Discussion

The central interpretive problem is that similar accuracy statistics can refer to different clinical questions. An AKI detection study, an analysis of persistent AKI, a comparison of active and inactive lupus nephritis, and a urinary tract infection study do not estimate the same diagnostic property. Population, specimen, collection time, reference definition, and threshold determine what each result means. The function–stress–injury framework helps organize these differences, but does not remove them [6,7,32,55].

The updated KIM-1 and NGAL syntheses change the estimates highlighted in earlier evidence summaries [14,15,16,23]; this reinforces the need to identify the source and setting of every numerical claim. A high AUC in a selected cohort does not establish superiority across diseases, and specificity in one study does not establish a universal confirmatory role. We therefore interpret biomarker measurements as complementary to clinical assessment, consistent with the ADQI consensus [55], without inferring absence of tubular injury from a negative result.

Cystatin C and PENK concern kidney function [25,28]; [TIMP-2]×[IGFBP7] concerns near-term AKI risk in specified settings [18,30,31]. KIM-1, NGAL, L-FABP, NAG, and interleukins provide injury-related or inflammatory information, although the clinical analyses differ [4,6,7,20]. CCL14 addresses persistence or non-recovery after AKI, with varying follow-up windows [3,34,35]. Acute-event uDKK3 estimates and associations with CKD progression likewise require separate outcomes and thresholds [8,19].

Disease-specific results need equally explicit boundaries. Urinary VCAM-1 was evaluated for lupus nephritis activity, and serum cathepsin S for albuminuria-defined DKD [36,37]. Urinary HBP indicates urinary tract infection, while plasma 8-isoprostane discriminates cross-sectionally for reduced eGFR in hypertension [40,42]. Neither should be grouped as direct confirmation of renal parenchymal injury. Regulatory status, where applicable, belongs to a defined assay and indication and does not establish equivalent clinical maturity for all protein biomarkers [33].

### 7.1. Prospective Evaluation of Combined Protein and ncRNA Strategies

We propose combining protein and ncRNA measurements as a research hypothesis based on their potentially complementary biological information. The studies summarized here do not establish an effective clinical sequence in which one marker identifies risk, another confirms injury, and an RNA signature resolves the diagnosis. Performance estimates from separate cohorts cannot demonstrate the incremental value of a combined panel. Evaluate a candidate combination against a prespecified clinical model with conventional measures in the same intended-use population, consistent with the need for context-specific biomarker evaluation [32,55]. Such studies should define the intended decision, sampling window, assay platform, and threshold before external validation. Evaluation should include discrimination, calibration (where a prediction model is used), and whether the result changes management or improves a patient-relevant outcome. A combined panel must also be feasible in terms of turnaround time, reproducibility, and cost. Protein assays differ in their degree of validation, and the ncRNA candidates reviewed remain at discovery or early validation stages.

### 7.2. Limitations

This is a purposive narrative synthesis with a targeted update, not an exhaustive systematic review or a new meta-analysis. Study-selection bias and incomplete coverage remain possible. No new formal study-level risk-of-bias scoring was performed. Differences in populations, reference definitions, sample handling, timing, thresholds, and analytical methods limit comparison across rows and sources. Historical and newer meta-analyses may include overlapping studies and should not be treated as independent replications. Some selected evidence concerns longitudinal association or GFR estimation rather than complete threshold-specific diagnostic accuracy. RNA studies may use small cohorts, healthy control groups, exploratory marker selection, or limited independent validation; these features constrain clinical generalizability. Mechanistic animal or cell studies do not validate human diagnostic tests. The source publications did not report accuracy measures, so we did not impute them; finally, the reported estimates do not establish a universal threshold, optimal panel, or incremental patient benefit.

## 8. Conclusions

Interpreting kidney biomarkers by intended use connects their biological information with clinically distinct questions. Cystatin C and PENK provide functional information; KIM-1, NGAL, L-FABP, and NAG provide tubular injury-related information; and [TIMP-2]×[IGFBP7] supports near-term AKI risk assessment in defined settings. CCL14 informs short-term persistence risk after AKI has developed, while uDKK3, VCAM-1, and cathepsin S address progression-related or disease-specific questions. HBP and 8-isoprostane describe complementary infection and oxidative-stress contexts. Emerging ncRNA findings extend this framework by providing candidate diagnostic signals and mechanistic evidence for injury and repair. Their combination with protein measurements is a plausible direction for prospective research, with clinical adoption dependent on independent validation and added value beyond conventional assessment.

## Figures and Tables

**Table 1:** Selected accuracy estimates for AKI detection or risk assessment.

Biomarker and Sample	Clinical Analysis	Sensitivity, % (95% CI)	Specificity, % (95% CI)	AUC (95% CI)	Interpretation	Reference
KIM-1 Urine	Adult AKI; 2025 meta-analysis.	73% (67–78)	75% (70–80)	0.81 (0.77–0.84)	Intervals labelled 95% CrI by the source. No universal cutoff or confirmatory role.	[15]
NGAL Urine	Pediatric AKI; 2023 meta-analysis.	83% (78–87)	81% (77–85)	0.89 (0.86–0.91)	Specimen, age, timing, and clinical setting constrain transferability.	[16]
PENK/penKid Circulating	AKI discrimination; meta-analysis.	69 (62–75)	76 (68–82)	0.77 (0.73–0.81)	Pooled results do not share one universal threshold.	[17]
[TIMP-2]×[IGFBP7] Urine	All-cause AKI risk; meta-analysis.	79 (72–84)	70 (62–76)	0.81 (0.78–0.84)	Mixed thresholds and settings; risk assessment does not identify the cause of injury.	[18]
uDKK3 Urine	AKI prediction; meta-analysis.	55 (41–68)	80 (70–87)	0.74 (0.70–0.77)	Primarily contrast-related and cardiac-surgery evidence; not a CKD progression estimate.	[19]
IL-18 Urine	Pediatric AKI; meta-analysis.	64% (32–87)	75% (62–85)	0.77 (0.74–0.81)	Wide sensitivity interval; distinct from serum IL-8.	[20]
IL-8/CXCL8 Serum		53 (34–72)	79 (60–91)	0.72 (0.68–0.76)	Different analyte and specimen from urinary IL-18.	

Abbreviations: AKI, acute kidney injury; AUC, area under the receiver operating characteristic curve; CI, confidence interval; CrI, credible interval; CXCL8, C-X-C motif chemokine ligand 8; IGFBP7, insulin-like growth factor-binding protein 7; IL, interleukin; KIM-1, kidney injury molecule-1; NGAL, neutrophil gelatinase-associated lipocalin; PENK, proenkephalin; TIMP-2, tissue inhibitor of metalloproteinases-2; uDKK3, urinary Dickkopf-related protein 3. Intervals are 95% CI except for KIM-1, for which the source uses the designation 95% CrI.

**Table 2:** Additional functional and tubular biomarker evidence.

Biomarker and Sample	Clinical Question	Evidence Summarized	Interpretive Limit	References
Cystatin C Serum	Assessment of glomerular filtration.	Clinical development and validation of GFR equations using cystatin C with creatinine.	Accuracy against measured GFR is not equivalent to AKI detection or direct evidence of tubular injury.	[25]
L-FABP/creatinine Urine	Prediction of hospital-acquired AKI in adults	2022 synthesis: HSROC 0.858 (95% CI, 0.749–0.938) for creatinine-normalized L-FABP; cardiac-surgery evidence also available.	Do not transfer this result to unnormalized L-FABP. Performance varies by population and sampling time.	[2,26]
NAG activity Urine	Adverse outcomes in established acute renal failure.	Prospective cohort, *n* = 201; association with dialysis requirement or in-hospital death.	Prognostic association; no universal early-AKI diagnostic threshold is inferred.	[27]

Abbreviations: AKI, acute kidney injury; CI, confidence interval; GFR, glomerular filtration rate; HSROC, hierarchical summary receiver operating characteristic; L-FABP, liver-type fatty acid-binding protein; NAG, N-acetyl-β-D-glucosaminidase.

**Table 3:** Prognostic and disease-specific applications.

Biomarker/Sample	Population and Outcome	Reported Result	Interpretation and Observations	Reference
CCL14 Urine	Established AKI; persistence or non-recovery.	2023 synthesis: Sensitivity 81% (72–87) Specificity 71% (53–84) AUC 0.84 (0.80–0.87) 2025 synthesis: AUC 0.79 (0.75–0.82)	Different evidence sets and follow-up windows; no common operating point.	[3,35]
uDKK3 Urine	CKD; subsequent short-term eGFR loss.	Longitudinal progression-related association.	Distinct from the acute-event AUC in Table 1; no AKI accuracy estimate is reused.	[8]
Soluble VCAM-1 Urine	Active versus inactive lupus nephritis.	Sensitivity 74.2% Specificity 74.2% AUC 0.84 (0.75–0.94) Cutoff >47.2 ng/mg creatinine	Sensitivity and specificity were identical. Separate confidence intervals for these estimates were not provided in Table 2 of the original study.	[36]
Cathepsin S Serum	Type 2 diabetes; UACR ≥30 versus <30 mg/g	Sensitivity 71.6% Specificity 98.8% AUC 0.900 (0.858–0.943) Cutoff 827.42 pg/mL	Cross-sectional, albuminuria-defined contrast; includes UACR > 300 mg/g.	[37]

Abbreviations: AKI, acute kidney injury; AUC, area under the receiver operating characteristic curve; CCL14, C-C motif chemokine ligand 14; CKD, chronic kidney disease; eGFR, estimated glomerular filtration rate; UACR, urinary albumin-to-creatinine ratio; uDKK3, urinary Dickkopf-related protein 3; VCAM-1, vascular cell adhesion molecule-1. Parentheses contain 95% confidence interval where reported.

**Table 4:** Markers associated with infection and nonprotein oxidative stress.

Marker/Sample	Clinical Contrast	Reported Accuracy	Interpretation	Reference
Infection-associated protein				
HBP-azurocidin Uridine	Symptomatic UTI versus asymptomatic controls; women ≥65 years.	Sensitivity 86% (75–93) Specificity 89% (82–94) AUC 0.92 Cutoff 16.7 ng/mmol creatinine	UTI discrimination does not establish renal parenchymal injury.	[40]
Nonprotein oxidative-stress analyte				
8-isoprostane Plasma	Hypertension; reduced eGFR <60 mL/min/1.73 m^2^.	Sensitivity 89% Specificity 89% AUC 0.931 Cutoff 329 pg/mL	Cross-sectional discrimination of reduced eGFR; prospective decline is not established by this analysis.	[42]

Abbreviations: AUC, area under the receiver operating characteristic curve; HBP, heparin-binding protein; eGFR, estimated glomerular filtration rate; UTI, urinary tract infection. Parentheses contain 95% confidence interval where reported.

**Table 5:** Selected RNA analyses and validation stages.

RNA Marker(s) and Sample	Clinical Analysis	Reported Result	Evidence Stage and Limitation	Reference
miRNA-5100 Serum	Human AKI versus healthy controls.	Sensitivity 100% Specificity 83.3% AUC 0.96 Expression cutoff 0.14	Exploratory human discrimination; companion mouse intervention. No pre-creatinine human detection established.	[45]
Ten serum miRNAs ^1^	ICU AKI versus healthy controls.	Individual AUCs 0.90–1.00.	Individual-marker pilot analyses. No common Sensitivity/Specificity or validated ten-marker classifier is assigned.	[46]
Four serum miRNAs ^2^	Preoperative prediction of AKI after cardiac surgery.	Marker- and cutoff-specific analyses.	Separate clinical question in the pilot study; the ICU diagnostic results are not transferred.	[46]
Four miRNAs and CSAG4 ^3^ Urine	CKD versus healthy controls; development.	Sensitivity 100% Specificity 92.5% AUC 0.9625 (0.9281–0.9969)	Combined model; CSAG4 is a pseudogene-associated transcript, not a fifth miRNA.	[47]
Same five-RNA combination ^3^ Urine	Separate set of 20 CKD patients and 20 controls.	Reported AUC 1.00 (1.00–1.00)	Small RT-qPCR set. External validation of a locked model is not clearly established.	[47]
miR-136-5p Urinary exosomes	DKD versus type 2 diabetes without DKD.	Sensitivity 72.2% Specificity 78.4% AUC 0.722 (0.552–0.892) Relative-expression cutoff 0.141	2025 clinical study; disease-specific discrimination, not prospective progression prediction.	[48]
RSDR Urinary sediment	69 patients with AKI versus 84 non-AKI controls.	Sensitivity 72.46% Specificity 72.62% AUC 0.7452	Individual RNA result. The internally validated multivariable model had a separate AUC of 0.864.	[49]

^1^ miR-101-3p, miR-127-3p, miR-210-3p, miR-126-3p, miR-26b-5p, miR-29a-3p, miR-146a-5p, miR-27a-3p, miR-93-3p, and miR-10a-5p. ^2^ miR-26b-5p, miR-27a-3p, miR-93-3p, and miR-127-3p. ^3^ miR-542-5p, miR-33b-5p, miR-190a-3p, miR-507, and CSAG4. Abbreviations: AKI, acute kidney injury; AUC, area under the receiver operating characteristic curve; CKD, chronic kidney disease; CSAG4, CSAG family member 4 (pseudogene); DKD, diabetic kidney disease; ICU, intensive care unit; miR/miRNA, microRNA; RNA, ribonucleic acid; RSDR, renal-specific defensive RNA; RT-qPCR, reverse transcription quantitative polymerase chain reaction. Parentheses contain source-reported 95% confidence interval.

## Data Availability

No new data were created or analyzed in this study. Data sharing is not applicable to this article. All information is derived from previously published studies cited in the reference list.
